# Essential concepts are missing: Foreign DNA in food invades the organisms’ cells and can lead to stochastic epigenetic alterations with a wide range of possible pathogenetic consequences

**DOI:** 10.1186/s13148-020-0813-z

**Published:** 2020-02-07

**Authors:** Walter Doerfler

**Affiliations:** 1 Institute for Clinical and Molecular Virology, Friedrich-Alexander University Erlangen-Nürnberg, Schlossgarten 4, 91054 Erlangen, Germany; 2 Institute of Genetics, University of Cologne, Cologne, Germany

**Keywords:** A new concept in general pathogenesis, Foreign DNA in mammalian cells and organisms, Epigenetic alterations upon foreign DNA intrusions, Origin of novel cell types, Uptake and persistence of food-ingested foreign DNA, Stochastic alterations of cell type, Relevance for pathogenesis of common human diseases

## Abstract

In this article, a new concept for general pathogenesis has been proposed. Advances in molecular genetics have led to the realization that essential concepts in the framework of molecular biology are still missing. Clinical medicine is plagued by similar shortcomings: The questioning of current paradigms could open new vistas and invite challenging approaches. This article presents an unconventional idea. Foreign DNA which is regularly ingested with the essential food supply is not completely degraded. Small quantities of fragmented DNA rather persist transiently in the gastro-intestinal tract of mice and can be traced to various organ systems, except for cells in the germ line. Foreign DNA entering and persisting in mammalian cells can stochastically lead to genome-wide alterations of transcriptional and CpG DNA methylation profiles. In the course of food-ingested DNA invading somatic cells, completely new cell types can be generated which might be involved in the causation of common ailments. Projects emanating from this perception merit critical analysis and rigorous pursuit.

## Introduction

Many of the common human diseases can be considered bad luck ailments [1] with uncertain genetic or anecdotal environmental causation. Stochastic reasoning just underscores the fact that we do not yet understand the hidden rules behind many phenomena and their pathogenesis. Traditional text book knowledge often fails to provide satisfactory, science-based explanations and tends to resort to detailed descriptions which medical practice has to rely on. In spite of remarkable insights into molecular genetic mechanisms with broad ramifications in medicine, in depth analytical exploration raises the suspicion that fundamental information in molecular biology is still lacking. More nucleotide sequence analyses and smart technicalities combined with refined statistical modeling may not lead us to the missing links. The intellectual situation today appears reminiscent of that of physicists and biologists in the 1930’s [2] who considered many avenues before critical experimentation led to the discovery of DNA as the universal carrier of genetic information [3].

In this article, two intertwined observations on the fate of foreign DNA in mammalian systems [4] and their combined impact on pathogenesis have become the basis for a new hypothesis.

1. The invasion of foreign DNA into mammalian cells, particularly in its genomically integrated configuration, can lead to genome-wide alterations of the cells’ transcriptional and DNA methylation profiles [4–8].
2. Fragments of foreign DNA, which are periodically taken up with the required food supply of mammals, survive transiently the passage through the gastro-intestinal (GI) tract. As documented in a mouse model [9, 10], these DNA fragments can be traced to peripheral blood mononuclear cells (PBMCs) and to cells in different tissues of the organism, except for cells in the germ line.
3. Based on and expanding from these two sets of experiment-based data, I submit that cells in an organism which are constantly and stochastically exposed to the intrusion of considerable amounts of food-derived foreign DNA will alter their function in the wake of altered transcription and CpG DNA methylation patterns. These alterations might become causative (co)factors for many of the common diseases, including neoplasias. Both events, fundamental changes in cell function and the occurrence of common diseases, have stochastic elements in common.

The essential experimental findings which support the author’s proposal will be briefly summarized in the following sections. The details of these data have been published [5–11].

## Intruding foreign DNA alters functional profiles of mammalian cells and generates different, possibly malignant cell types

### Integrated viral genomes

In a hamster cell line transgenomic for about twelve copies of chromosomally integrated human adenovirus type12 (Ad12) DNA, the levels of cellular CpG DNA methylation are altered genome-wide [5]. Similar findings have been reported in hamster cells transgenomic for bacteriophage lambda DNA, although to a lesser extent [6]. Moreover, the transcriptional profiles of cellular DNA in hamster cells transgenomic for Ad12 DNA or lambda DNA have changed [7]. In a revertant of one of the Ad12 transformed cell lines, which has lost all of the Ad12 transgenomes, the increase of genome-wide DNA methylation in intracisternal A particle (transposon) DNA persisted even in the absence of the transgenomic Ad12 DNA. Hence, in this instance, altered DNA methylation profiles proved inheritable and were likely initiated by a hit-and-run mechanism. The guard for the stability of the methylation profile had been called upon by Ad12 DNA integration, remained alerted after the loss of foreign DNA, and was stably transmitted to the next cell generations [5, 8].

### Small bacterial plasmid

A different experimental scenario was devised to challenge the stability of cellular transcriptional and DNA CpG methylation profiles in individual cell clones of human colon tumor (HCT116) cells upon the insertion of a 5.6 kbp bacterial plasmid. Single-cell clones were generated which contained either no transgenome or the 5.6 kbp plasmid DNA which carried the kanamycin resistance gene for clonal selection as sole transcribed element. In the subsequently expanded cell clones, differences in transcriptional and CpG methylation patterns between transgenomic and non-transgenomic cell clones were determined. Transcriptional and CpG methylation profiles of all transcripts investigated in five clonal cell populations of non-transgenomic controls were almost identical. This finding facilitated comparisons of the differential gene segment expression between non-transgenomic and transgenomic cell clones in 28,869 genomic DNA segments: 1343 showed differential expression with 907 regions up- and 436 regions downregulated [11]. Thus, the integration of the 5.6 kbp bacterial plasmid into the human genome altered transcription profiles in 4.7% of the investigated gene segments. The functional details about the DNA segments differentially transcribed and further information on experimental procedures have been published earlier [11].

Furthermore, when CpG DNA methylation profiles in the same sets of non-transgenomic versus 5.6 kbp plasmid-transgenomic HCT116 cell clones were assessed in 361,983 CpG dinucleotide positions, 3791 CpGs were found differentially methylated in the transgenomic cell clones, and 1504 proved hyper- and 2287 hypo-methylated. Hence, both transcription and CpG methylation profiles were altered in human cells by genomically inserting a 5.6 kbp DNA plasmid [11]. All the data in this experimental setting were statistically verified by Benjamini-Hochberg corrections [11, 12] to exclude false-positive values.

### Episomal persistence of EBV genomes

In a different biological system a similar observation was made on changes in DNA methylation patterns in cells which carried foreign DNA. One of the most frequently occurring forms of mental retardation in humans is the Fragile X Syndrome (FXS) [13]. It is molecularly characterized by the expansion of a naturally occurring CGG repeat in the untranslated first exon of the human *fragile X mental retardation gene 1 (FMR1)* and the CpG hyper-methylation in its promoter region. In the region upstream of this promoter, we have identified a distinct boundary between unmethylated genome segments, as they exist in unaffected individuals, and DNA sequences further upstream from the promoter which are strongly CpG methylated. In FXS individuals, the methylation boundary is obliterated; the promoter becomes CpG hyper-methylated and inactivated. As a consequence, the *FMR1* gene product fails to be produced; its lack during an individual’s development leads to the FXS syndrome. The methylation boundary is thought to exhibit a specific chromatin structure which demarcates a hyper-methylated upstream sequence from the unmethylated downstream *FMR1* promoter. The boundary sequence, as it were, protects the promoter from the spreading of DNA methylation and thus helps maintain its activity [14].

In normal human PBMCs, the far upstream genome region beyond the boundary is hyper-methylated. In PBMCs immortalized by transformation with *EBV* or with the human *telomerase* gene, the boundary is maintained. However, the region far upstream from the boundary becomes hypo-methylated by a factor of 4 [15] in PBMCs both from non-FXS and from FXS individuals. These specific alterations in methylation levels likely are caused by the episomally persisting foreign *EBV* DNA genome or the integrated *telomerase* gene [8, 15]. Destabilization of cellular methylation patterns has also been reported in human lymphoblastoid cell lines after transformation by *EBV* [16].

Although many questions remain as to mechanisms involved and the generality of alterations in transcriptional and CpG DNA methylation profiles, the ensemble of data presented raises the question of to what extent the introduction of foreign DNA into mammalian cells destabilizes the epigenetic landscapes in mammalian cells. By the same token, due to the mentioned epigenetic sequelae of foreign DNA intrusion, functionally completely new cell types arise and likely impact on the mechanisms causing common diseases. The stochastic nature of disease incidence is paralleled by the stochasticity of the molecular events linked to foreign DNA intrusion and intranuclear fixation.

## DNA fragments from the food supply survive transiently the passage through the gastro-intestinal tract and can intrude into somatic cells of the organism

In a mouse model, we initially followed orally administered bacteriophage M13mp18 DNA to determine the fate of food-ingested foreign DNA in a mammalian organism. Fragments of this DNA were detectable in all parts of the gastro-intestinal (GI) tract of mice between 18 and 24 h after feeding (*n* = 84). Between 2 and 8 h after feeding, fragments of up to 1.700 bp were retrieved in PBMCs, and in spleen or liver cells up to 24 h after feeding (*n* = 254) both by PCR and fluorescent in situ hybridization (FISH) [9]. About 1 to 2% of the orally administered DNA could be retrieved as fragments in different parts of the GI tract. Fragments of about 700 bp of the fed DNA were detected both by PCR and FISH in different types of peripheral white blood cells. Adequate negative controls with either method corroborated the results. Upon long-term feeding, the test DNA could be recloned from total spleen DNA. In one of the phage lambda vector clones, a 1.300 bp M13mp18 DNA fragment was documented to have become covalently linked to DNA with 70% homology to the mouse *IgE receptor* gene [9]. The frequency of possible genomic integration of food-ingested DNA into the cellular genome of spleen cells will have to be further investigated to certify the generality of this event.

In an extension of these studies, soybean leaves were fed to mice, and the distribution of the plant-specific, nucleus-encoded *ribulose-1,5-bisphosphate carboxylase (Rubisco)* gene was assessed in the mouse organism and found in different parts of the GI tract, in spleen and liver DNA [10]. Transcription of the persisting *Rubisco* gene could not be detected. Additionally, mice were continuously fed plasmid DNA carrying the cloned *green fluorescent protein (GFP)* gene for eight generations. Subsequently, DNA from tail tips or internal organs of the offspring was examined by PCR in each of the eight generations for germ-line transmission of orally administered *GFP* DNA. Results were consistently negative arguing against germ-line entry of orally fed foreign DNA. In controls, *GFP* DNA administered by intramuscular injection was detected in several organ systems remote from the injection site [10].

This ensemble of data supports the conclusion that orally administered foreign DNA persists upon passage through the GI tract and reaches several organ systems of the test animals. Transcripts of the foreign DNA were not detectable. Foreign DNA continuously administered orally over eight generations of mice had not penetrated into their germ lines. We pursue the possibility that DNA from the food supply manages to enter the murine organism and can be recovered in fragmented form from several organ systems, except from cells in the germ-line.

## Synopsis and data-derived hypothesis

Two major results have been reported:

1. Foreign DNA when introduced into mammalian cells in the episomal or chromosomally integrated state can elicit genome-wide alterations of the cell’s transcriptional and DNA CpG methylation profiles [4–8]. Potentially, completely new cell types will arise under this regimen.
2. There is a constant flow of foreign DNA fragments derived from the broad spectrum of food-ingested DNA which can be distributed to many cells in mammalian organisms with the exception of germ cells [9, 10]. It is uncertain to what extent, for how long, and in which of the two modes—integrated or episomal—these DNA fragments will be able to persist in the recipient’s cells. It is tempting to combine these data-based insights from experimental work and pursue the notion that somatic cells affected by foreign DNA intrusion can be transformed in a stochastic mode to functionally different cell types. Of course, the organisms’ surveillance systems will be constantly screening for these epigenetically altered cells. As a consequence, many of these “transformed” cells may fall prey to existing defense mechanisms. With increasing age of individuals and under their specific life-long stress experiences, altered or transformed cells will survive stochastically and expand in number, particularly when growth control functions have been compromised by foreign DNA intrusions. In this context, the impact on the broad range of common diseases and their pathogenesis will have to be considered: Malignancies, autoimmune diseases, metabolic disorders, cardio-vascular diseases, and others may fall into this category. There is evidence from one of our projects [5] that the stability of altered CpG methylation patterns is not dependent on the continued presence of the causative foreign DNA. Apparently, changes of CpG methylation can be elicited by a hit-and-run mechanism.
3. Hypothesis: Food-ingested DNA is capable of intruding into diverse somatic cell types, which fundamentally alters these cells and thus contributes to general pathogenetic mechanisms in the organism. In contrast to plant DNA, DNA from animal-derived food might have higher pathogenetic potential since mammalian DNA likely can more frequently recombine with human DNA due to its closer evolutionary relatedness to human DNA.

The same wake-up call can be raised for the consequences of all types of genome manipulations, e.g. by regimens of gene therapeutic approaches, by knock-out or the CRISPR-Cas9 strategies when applied directly to human organisms of all ages. Many of these approaches involve the import of foreign DNA into cells and manipulations at the genome level. At this stage, direct experimental evidence has not yet been adduced for the above-elaborated mechanisms to be operative in pathogenesis. However, an argument can be made that such evidence has not been sought vigorously enough, particularly in gene technologically altered cells.

## Prospects and experimental approach

It will not be feasible to verify this unconventional concept directly in a one-step approach. I opt for proceeding with a stepwise strategy. Currently, the following goals are being pursued:

It will be cogent to revisit the project of following food-ingested DNA in mice with improved technology which was not available 20 plus years ago [9, 10]. In the current experimental plan, plant-specific genes and/or DNA segments will be exclusively used as test DNAs to avoid sequence ambiguities with mammalian DNA. Moreover, the DNA constructs fed orally to mice will carry genetically selectable markers to facilitate their re-cloning from the DNA of host organs. In addition, fluorescence tags will be imposed on the test DNA to allow for direct detection in host tissues by state-of-the-art microscopy. Cells from many different tissues from foreign DNA-fed mice will be examined by these procedures. The germ cells from these animals will also be re-investigated, although previous work [10] had shown the germline to be protected from the intrusion of food-ingested DNA.

In addition, experiments will be repeated in which we succeeded in molecularly re-cloning food-ingested DNA fragments from the host’s spleen DNA and in determining the abutting host DNA from the integration site [9]. The presence of a genetically selectable marker, like the kanamycin resistance gene, in the test DNA to be orally administered to mice should enhance the chances for successfully re-cloning the test DNA and its integration site.

## Data Availability

Of course, I will make data and materials available to interested colleagues.

## Abbreviations

Ad12: Human adenovirus type 12
bp: Base pair(s)
CCG: Cytosine-cytosine-guanosine trinucleotide
CpG: Cytosine-guanosine dinucleotide
DNA: Deoxyribonucleic acid
EBV: Epstein-Barr virus
*FMR1*: *Fragile X Mental Retardation 1*
FXS: Fragile X Syndrome
*GFP*: *Green fluorescent protein*
GI: Gastro-intestinal
HCT116: Human Colon Tumor 116
IgE: Immune globulin E
kbp: Kilo base pairs
M13mp18: Designation of a bacteriophage (M for Munich, 13 protocol number)
PBMCs: Peripheral Blood Mononuclear Cells
PCR: Polymerase Chain Reaction
Rubisco: *Ribulose-1,5-bisphosphate carboxylase*
